# Membrane Separation for the Treatment of LiBr + LiCl Brines and Their Application

**DOI:** 10.3390/membranes15080219

**Published:** 2025-07-23

**Authors:** Jonathan Ibarra-Bahena, Ulises Dehesa-Carrasco, Yuridiana Rocio Galindo-Luna, Iván Leonardo Medina-Caballero, Wilfrido Rivera

**Affiliations:** 1 Instituto de Energías Renovables, Universidad Nacional Autónoma de México, Privada Xochicalco S/N, Temixco, Morelos 62580, Mexico; jibarra@ier.unam.mx (J.I.-B.); mecai@ier.unam.mx (I.L.M.-C.); 2 El Colegio de Chihuahua, Partido Díaz 4723, Cd. Juárez, Chihuahua 32310, Mexico; udehesa@colech.edu.mx; 3 Departamento de Ingeniería de Procesos e Hidráulica, Universidad Autónoma Metropolitana-Iztapalapa, Av. Ferrocarril San Rafael Atlixco 186, Col. Leyes de Reforma 1 A Sección, Iztapalapa, Ciudad de México 09310, Mexico; ygalindol@izt.uam.mx

**Keywords:** air gap membrane distillation, sorption cooling systems, H_2_O/LiBr + LiCl solution, brines

## Abstract

In sorption cooling systems, an important stage of the thermodynamic cycle is the separation of the refrigerant fluid from the absorbent mixture. This process is called “regeneration” or “desorption,” and it is similar to thermal desalination, where water is separated from an aqueous saline solution. However, since sorption systems utilize high salt concentration solutions, conventional desalination techniques such as reverse osmosis are not suitable. In this regard, membrane devices can enhance heat and mass transfer processes in compact sizes. In the present paper, a membrane device with an air gap membrane distillation configuration was evaluated, operating with the H_2_O/LiBr + LiCl solution (with a mass ratio of 2:1, LiBr:LiCl), to assess the produced distilled water flux. Among the operating parameters analyzed (solution temperature, cooling water temperature, salt concentration, and membrane pore size), solution temperature had the highest impact on the distilled water flux, while the membrane pore size had the lowest impact. The maximum distilled water flux was 7.63 kg/h·m^2^ with a solution temperature of 95.3 °C, a cooling water temperature of 25.1 °C, a salt concentration of 44.99% *w*/*w*, and a membrane pore size of 0.45 μm. On the other hand, the minimum distilled water flux was 0.28 kg/h·m^2^ with a solution temperature of 80.3 °C, a cooling water temperature of 40.1 °C, a salt concentration of 50.05% *w*/*w*, and with a membrane pore size of 0.22 μm.

## 1. Introduction

Thermally driven systems related to thermal comfort (such as liquid desiccant–air contact and absorption chillers) have been widely studied, as they offer promising and eco-friendly alternatives to conventional vapor compression devices, and can help reduce global warming. In addition, neither chlorofluorocarbons (CFCs) nor hydrochlorofluorocarbons (HCFCs) are used as working fluids, nor are fossil fuels used as a heat source, and relatively low electricity consumption is required [1]. In this technology, an important step is the separation of the refrigerant fluid from the absorbent mixture. This process is called “regeneration” or “desorption,” and it is similar to thermal desalination, where water is separated from an aqueous saline solution. The H_2_O/LiBr (water–lithium bromide) solution is the most used saline solution for brine–water sorption cooling systems; however, LiBr crystallization reduces the operational temperature range [2]. Increasing the LiBr solubility means the crystallization risk is reduced; in this regard, the use of additives such as organic salts [3,4,5], alcohols [6,7,8,9,10], ionic liquids [11,12,13], or inorganic salts [14,15,16] has been proposed and analyzed in the literature. To widen the operating range of a sorption cooling system, a working mixture with a low crystallization temperature is desirable [17], and the use of hygroscopic salts can help achieve this condition. Increasing the absorbent salt concentration in the working mixture reduces the water vapor pressure, which improves the refrigeration process efficiency [11]. Lithium chloride (LiCl) is a common absorbent used in sorption cycles. Additionally, it is less expensive than LiBr and exhibits long-term stability and good hygroscopic properties [18]. Therefore, the advantages of individual absorbents (LiBr and LiCL) can produce an interesting ternary mixture (H_2_O/LiBr + LiCl) for sorption cooling cycles. This mixture was proposed and analyzed by Patil et al. [19], who used a mixture with a mass ratio of 1:1 (LiBr:LiCl) in an absorption heat pump. The authors noted the advantages of this ternary mixture, which includes lower corrosivity due to the addition of LiCl, reducing the amount of LiBr. Consequently, the solubility of the absorbent salts increases, thereby reducing the crystallization risk, and the solution cost is lower than that of the conventional H_2_O/LiBr mixture. According to the authors’ results, the use of a ternary mixture can enhance the interaction between the working fluid and the absorbent. Grover et al. [20] used the same ternary mixture in an absorption cooler. The authors concluded that the generator heat load was lower with the ternary mixture than with the H_2_O/LiCl mixture. Pataskar et al. [21] evaluated an absorption heat transformer operating with the H_2_O/LiBr, H_2_O/LiBr + LiCl (with a mass ratio of 1:1, LiBr:LiCl), and the H_2_O/LiCl mixtures. According to the authors’ results, the Coefficient of Performance (COP) values for the ternary mixture were higher. However, the temperature lifts were lower compared to those for the H_2_O/LiBr mixture under similar operating conditions. Recently, Arabi and Dehghani [22] proposed the H_2_O/LiBr+NaHCO_2_ and H_2_O/LiBr + LiCl mixtures (with a mass ratio of 2:1) as alternatives for absorption cooling systems. The authors reported the solubility and density, and according to their results, the solubility of the H_2_O/LiBr + LiCl mixture is higher. In contrast, the density is lower than the conventional H_2_O/LiBr mixture. Based on these results, Aktemur and Öztürk [23,24] carried out an energy analysis of a solar-driven absorption cooling system with 50 kW of cooling capacity. The authors concluded that, compared with the H_2_O/LiBr mixture, the H_2_O/LiBr + LiCl mixture increased the COP and exergy efficiency by 8.81% and 8.96%, respectively. Meanwhile, the circulation ratio, solar collector area, and storage tank volume were reduced by 48.93%, 8.92%, and 8.91%, respectively. From the literature reviewed, it is clear that the H_2_O/LiBr + LiCl mixture is a viable alternative for sorption systems since several advantages have been reported.

Besides the impact of the selected working mixture on the sorption device’s performance, compact components are desirable for small-scale applications. Typically, the generator or regenerator used in sorption heat pumps is large and heavy; additionally, the heat and mass transfer of these key components are deficient, and the capital costs are higher than those of vapor compression systems [25,26]. Since sorption systems use high salt concentration solutions, conventional desalination techniques such as reverse osmosis (RO) are not suitable. Therefore, water separation is carried out by boiling at low operating pressure, which requires auxiliary devices. In this regard, membrane separation technologies, such as membrane distillation (MD), are emerging alternatives for treating high salt concentration solutions. In membrane distillation (MD), the water in the vapor phase is separated from a saline solution due to the vapor pressure difference caused by a temperature difference. Since this desorption process occurs below the boiling point of the working mixture, low-grade heat sources are suitable [27]. MD modules can enhance heat and mass transfer processes in reduced sizes, as the area-to-volume ratio is higher than that of conventional boiling components [25]. Several authors have reported the application of membrane devices in sorption heat pumps [25,28,29].

In this paper, an experimental evaluation of a membrane device using the H_2_O/LiBr + LiCl ternary mixture (with a mass ratio of 2:1, LiBr:LiCl) is presented and compared with other working mixtures reported in the literature. The effect on the distilled water flux of operation parameters such as salt concentration, pore size, saline solution temperature, and condensation temperature, was analyzed. The membrane configuration used was air gap membrane distillation (AGMD), as it achieves two purposes (desorber or regenerator/condenser) with this configuration.

## 2. Materials and Methods

### 2.1. Membrane Device Description

**Figure 1:**
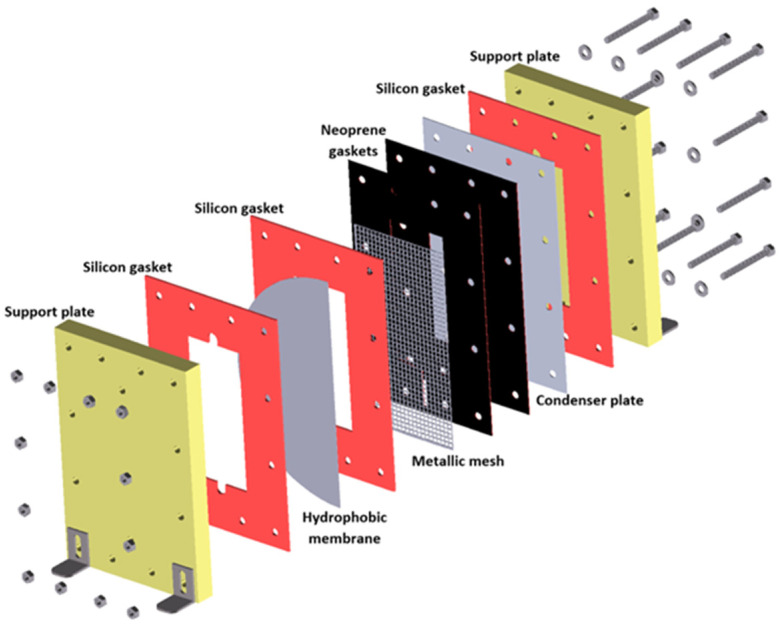
Experimental membrane device configuration.

The membrane device has been described in previous reports [30,31] and comprises two support plates made of a polymeric material 300 mm in length, 200 mm wide, and 25.4 mm thick; neoprene gaskets and heat-resistant silicon gaskets with 1 and 3 mm thicknesses, respectively; a metallic mesh to support the membrane to reduce the deformation of the membrane by the solution flow; an aluminum plate with a 0.4 mm thickness for vapor condensation; and 12 bolts and screws. The solution channel was created by the junction of the support plate, the heat-resistant silicon gasket, and the membrane, measuring 180 mm in length, 80 mm in width, and 3 mm in thickness. The air gap, with a thickness of 3 mm, was integrated by the opposite membrane face, two neoprene gaskets, a metallic mesh, one silicon gasket, and one side of the condensing plate. Finally, the cooling water channel included the posterior side of the condenser plate, the heat-resistant silicon gasket, and the second support plate. Polytetrafluoroethylene (PTFE) hydrophobic membranes were used. Due to the high porosity and high hydrophobicity of the PTFE membranes, they are suitable for the MD process. In addition, the permeate flux is higher than that of the polyvinylidene difluoride (PVDF) and polypropylene (PP) membranes in the AGMD configuration [32]. Figure 1 shows the components of the experimental membrane device.

### 2.2. Experimental Setup

**Figure 2:**
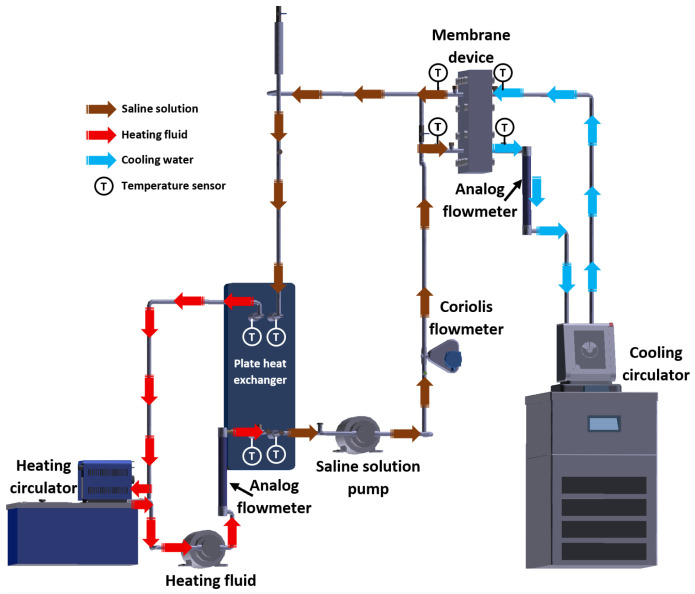
Experimental setup diagram.

The experimental setup was integrated by the membrane device, heating and cooling systems, and the instrumental devices required to quantify the operating parameters. The heat load required for the membrane distillation process was supplied by a heating circulator with temperature control (by Julabo, Seelbach, Germany), which heated a fluid used to transfer sensible heat to the saline solution inside a plate heat exchanger (PHE) manufactured by Alfa Laval (Lund, Sweden). The latent heat load produced by the condensation of water in the vapor phase on the cooling plate was removed by cooling water refrigerated with a cooling circulator that features an integrated pump and temperature control (by Julabo, Seelbach, Germany). A Coriolis mass flowmeter (by Emerson Electric Co., St. Louis, MO, USA) was used to measure the mass flow of the saline solution, while the heating fluid stream and the cooling water stream were measured with analog flowmeters (by Cole Parmer, Vernon Hills, IL, USA). The solution and the heating fluid were pumped using gear pumps (by Cole Parmer, Vernon Hills, IL, USA) with a power rating of 32 W. An electronic weighing scale (by Optima Scale, Rancho Cucamonga, CA, USA) was used to measure the weight of the distilled water. The inlet and outlet temperatures of the membrane device and the PHE were measured with RTD pt100 temperature sensors (by Omega Engineering, Stamford, CT, USA). An Agilent data acquisition unit (by Agilent Technologies, Santa Clara, CA, USA) recorded the temperatures and the mass flow data of the solution. The H_2_O/LiBr + LiCl solution was prepared by mixing an aqueous LiBr solution with a concentration of 54% *w*/*w* and a density of 1.57 kg/L (at 25 °C), and crystalline LiCl powder, both chemical compounds were provided by Sigma Aldrich (Burlington, MA, USA). Since the mass ratio was 2:1 (LiBr:LiCL), to prepare 1 kg of saline solution with a concentration of 45% *w*/*w*, 0.556 kg of LiBr solution was mixed with 0.150 kg of LiCl and 0.294 kg of distilled water. To prepare 1 kg of saline solution with a concentration of 50% *w*/*w*, 0.333 kg of LiBr solution, 0.167 kg of LiCl, and 0.216 kg of distilled water were required. The salt concentration was calculated with the correlation reported by Arabi and Dehghani [22]. Table 1 presents the uncertainties associated with the devices used to measure the operating variables in the experimental tests. The experimental setup is shown in Figure 2.

### 2.3. Experimental Operating Conditions

Different solution temperatures, salt concentrations, cooling water temperatures, and membrane pore sizes were used to analyze their effects on the distilled water flux. Table 2 shows the experimental operating conditions.

## 3. Results

To evaluate the membrane device performance with the H_2_O/LiBr + LiCl mixture, the saline solution and cooling water temperatures were varied, together with the salt concentration and the membrane pore size. The results are present in the following subsections.

### 3.1. Operation Temperatures

**Figure 3:**
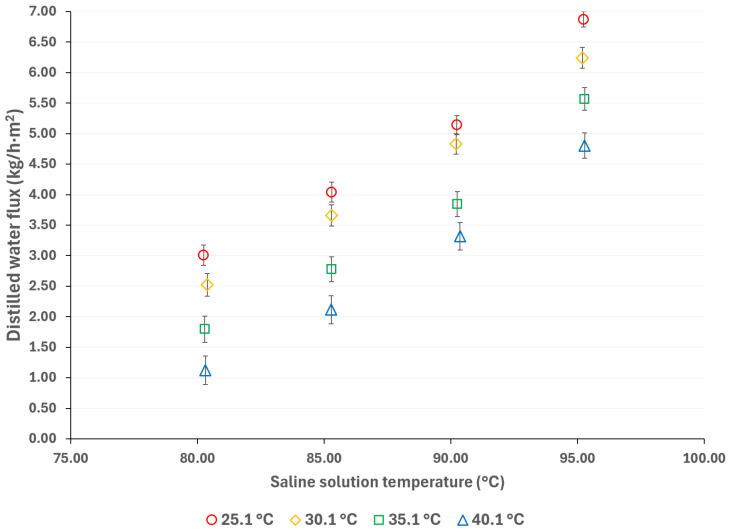
Distilled water flux for different operating temperatures with a membrane pore size of 0.22 μm.

**Figure 4:**
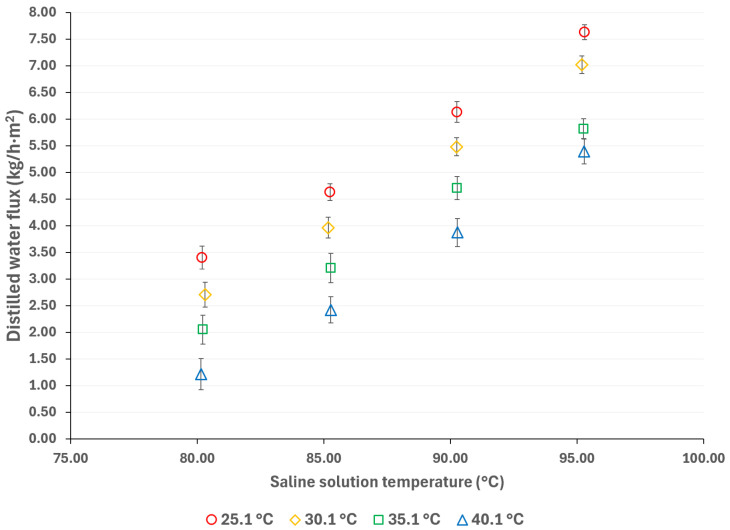
Distilled water flux for different operating temperatures with a membrane pore size of 0.45 μm.

To mitigate the effects of global warming, utilizing renewable energy sources for cooling sorption systems is desirable. Since solar energy is suitable for driving thermal separation processes such as MD, the salt solution temperatures were selected based on a possible scenario involving the integration of thermal solar technologies [33]. Additionally, if waste heat is utilized as a complementary thermal source (which is relatively inexpensive), an energy cost reduction can be achieved [34]. The cooling water temperatures were selected considering a wide environmental temperature range, allowing for the convenient selection of auxiliary systems to remove the heat load in the condenser. Figure 3 and Figure 4 show the distilled water flux for membrane pore sizes of 0.22 μm and 0.45 μm, respectively, with a salt concentration of 44.99% *w*/*w* and the operation temperatures shown in Table 1. In these figures, the effect of the operational temperatures can be observed. The impact of the saline solution temperature on the distilled water flux is higher than the cooling water temperature. For instance, in Figure 3, with a membrane pore size of 0.22 μm, increasing the solution temperature from 80.3 to 95.3 °C at a cooling water temperature of 25.1 °C results in a 128% increase in the distilled water flux. On the other hand, by decreasing the temperature of the cooling water from 40.1 °C to 25.1 °C, at a solution temperature of 95.3 °C, the distilled water flux increases by 43%. Similar behavior is observed in Figure 4, where a membrane pore size of 0.45 μm results in an increase in distilled water flux of 124% and 41% when the solution temperature increases from 80.3 to 95.3 °C and the cooling water temperature is reduced from 40.1 to 25.1 °C, respectively. Since membrane distillation is a thermally driven separation process, the temperature difference between the hot and cold sides of the membrane increases the distilled water flux because the vapor partial pressure at the membrane interphase increases exponentially with the solution temperature [35]. Additionally, the viscosity of the saline solution decreases as the solution temperature increases, which reduces the thickness of the boundary layer at the membrane, thereby enhancing the mass transfer coefficient [36]. Because there is no information about the viscosity and vapor pressure of the analyzed mixture, to illustrate the effect of the solution temperature on viscosity and the vapor mass transfer, the reported data with the H_2_O/LiBr solution with a salt concentration of 49.96% *w*/*w* was used to make an analogy. For this solution, the contribution of the boundary layer mass transfer resistance, which is related to the solution viscosity, was reduced by 22% when the solution temperature increased from 75.2 °C to 95 °C [37].

### 3.2. Membrane Pore Size

**Figure 5:**
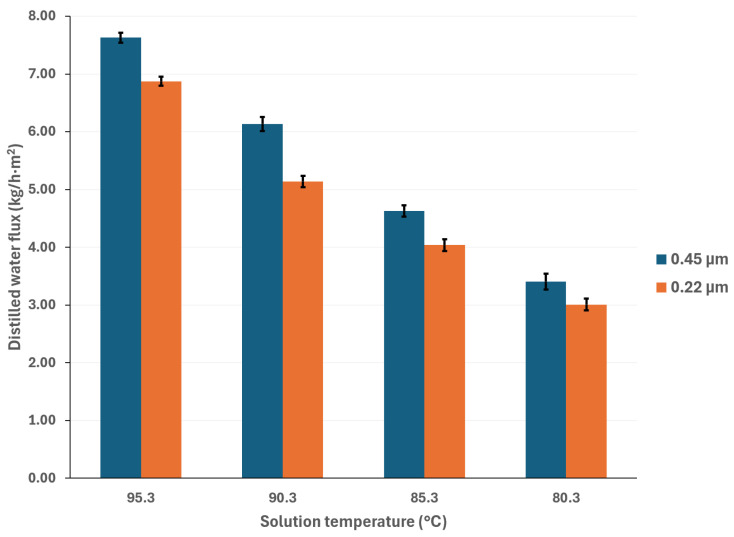
Distilled water flux comparison between membrane pore sizes of 0.22 μm and 0.45 μm, with salt concentration of 44.99% *w*/*w* and cooling water temperature of 25.1 °C.

**Figure 6:**
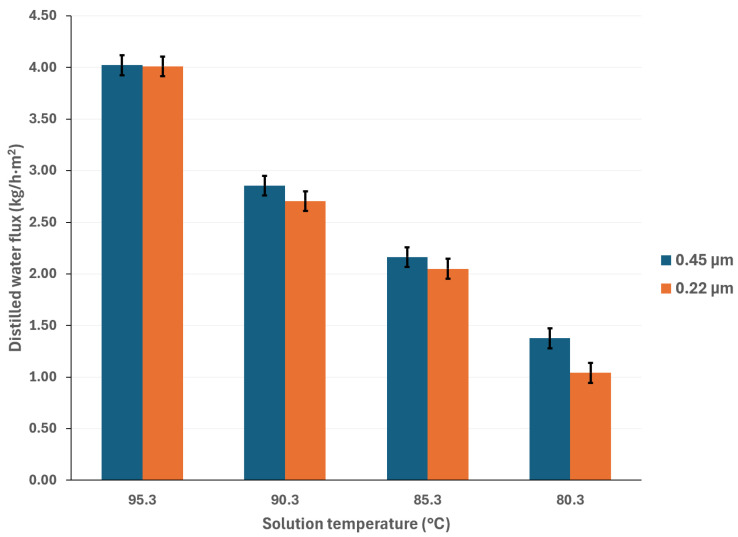
Distilled water flux comparison between membrane pore sizes of 0.22 μm and 0.45 μm, with salt concentration of 50.05% *w*/*w* and cooling water temperature of 25.1 °C.

Generally, higher membrane pore sizes are desirable since they improve mass transfer and reduce heat transfer by conduction; however, as the membrane pore size increases, the liquid entry pressure (LEP) decreases, which can lead to membrane wetting and solute contamination of the distilled water [38,39]. Figure 5 compares the distilled water flux with membrane pore sizes of 0.22 µm and 0.45 µm, at a cooling water temperature of 25 °C, and with a salt concentration of 44.99% *w*/*w*. Figure 6 shows the same comparison with a salt concentration of 50.05% *w*/*w*. It is expected that, as the pore size increases, the distilled water flux will also increase; however, this behavior is observed at the lowest salt concentration. According to Khalifa et al. [40], the increase in distilled water flux is not a linear function of the pore size. In addition, the vapor partial pressure, which is independent of membrane pore size, is mainly influenced by the temperature and salt concentration, thus, the effect of these parameters on the mass transport driving force is greater than that of the membrane pore size [41]. For instance, with a solution temperature of 95.3 °C and cooling water at 25.1 °C, the distilled water flux increased 11% when using a membrane with a pore size of 0.45 μm compared to a membrane with a pore size of 0.22 μm, with the lower salt concentration. On the other hand, with the highest salt concentration, at the same operation temperatures, the distilled water flux difference between the two pore sizes is negligible (0.3%). This effect has been reported by other authors in high salt concentration solutions [41,42,43].

### 3.3. Salt Concentration

**Figure 7:**
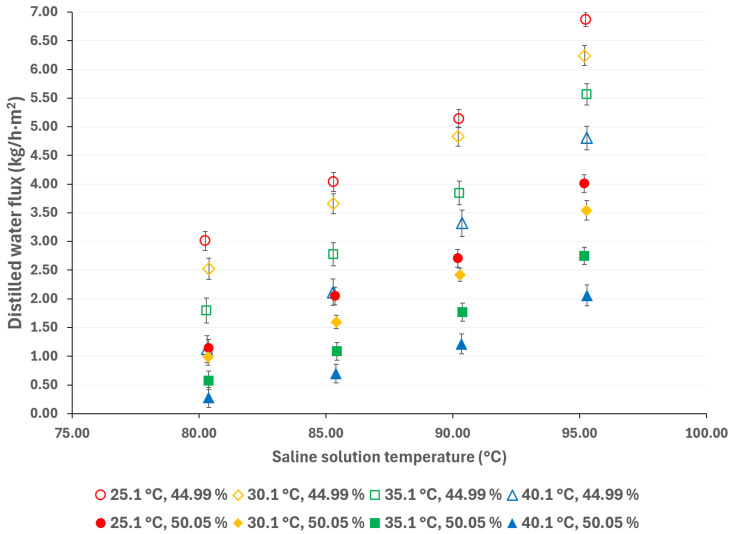
Distilled water flux for different operating conditions with a membrane pore size of 0.22 μm.

**Figure 8:**
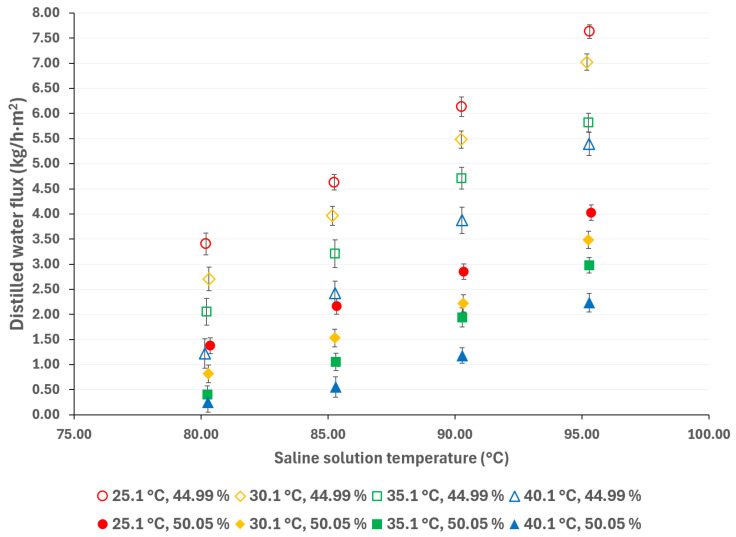
Distilled water flux for different operating conditions with a membrane pore size of 0.45 μm.

Compared to pressure-driven desalination technologies (such as RO), AGMD is more suitable for treating high-salinity solutions; however, thermodynamic properties, including vaporization heat, surface tension, boiling point, and viscosity, increase with salt concentration, thereby affecting the flux of the distilled water [44]. The influence of salt concentration can be observed in Figure 7 and Figure 8. As expected, a decreasing salt concentration increases the distilled water flux. According to the results presented in Figure 7, at a solution temperature of 95.3 °C and a cooling water at 25.1 °C, the distilled water flux increased by 71%, resulting in a decrease in salt concentration from 50.05% *w*/*w* to 44.99% *w*/*w*. Similarly, as shown in Figure 8, the distilled water flux increased by 90% at similar operation temperatures.

According to the results presented in Figure 3, Figure 4, Figure 5, Figure 6, Figure 7 and Figure 8, the operation parameters that improve the distilled water flux, in order of importance, are as follows: the solution temperature, the salt concentration, the cooling water temperature, and, ultimately, the membrane pore size.

### 3.4. Comparison with Respect to the Conventional H_2_O/LiBr and H_2_O/LiCl Solutions

As previously mentioned, H_2_O/LiBr and H_2_O/LiCl are the most commonly used saline solutions in sorption systems. Thus, a performance comparison was conducted based on previous results available in the literature, using similar operating conditions as described in Table 2 and the same membrane device. Table 3 and Table 4 present the experimental conditions reported with the H_2_O/LiBr and H_2_O/LiCl solutions, respectively.

**Figure 9:**
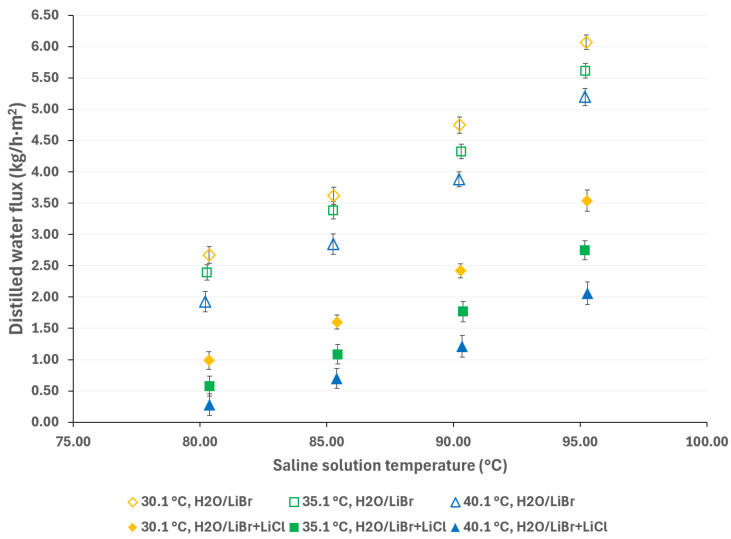
Distilled water flux comparison between the H_2_O/LiBr and H_2_O/LiBr + LiCl solutions.

In Figure 9, the distilled water flux of the H_2_O/LiBr and H_2_O/LiBr + LiCl solutions is presented; the empty markers are for the H_2_O/LiBr saline solution, while the solid markers are for the H_2_O/LiBr + LiCl saline solution. As can be seen, under similar operating conditions, the performance of the H_2_O/LiBr solution is superior to that of the H_2_O/LiBr + LiCl solution. For instance, at a solution temperature of 95.3 °C and a cooling water temperature of 30.1 °C, the distilled water flux is 71% higher.

**Figure 10:**
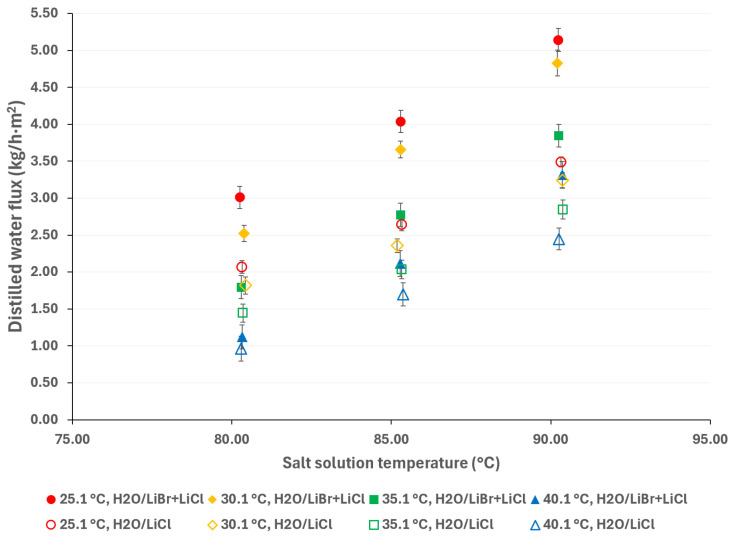
Distilled water flux comparison between the H_2_O/LiCl and H_2_O/LiBr + LiCl solutions.

On the other hand, according to Figure 10, the distilled water flux with the H_2_O/LiBr + LiCl solution is 38% higher than that with the H_2_O/LiCl solution, with a saline solution temperature of 90.3 °C and a cooling water temperature of 25.1 °C. In this figure, the empty markers represent the H_2_O/LiCl saline solution, while the solid markers represent the H_2_O/LiBr + LiCl saline solution.

Even though the solution mass flow used with the H_2_O/LiCl solution was lower than reported in Table 1, this condition slightly impacts the distilled water flux [45] and, based on the data reported by Ibarra-Bahena et al. [31], increases the solution mass flow from 3.50 × 10^−2^ to 4.00 × 10^−2^ kg/s, with H_2_O/LiBr only increasing the distilled water flux by 3%. In addition, the salt concentration of the H_2_O/LiBr + LiCl solution was higher (44.99% *w*/*w*) than that of the H_2_O/LiCl solution (41.05% *w*/*w*).

According to Chen and Xu [46], the diffusion coefficient for water molecules in the H_2_O/LiBr solution is around 40% higher than that in the H_2_O/LiCl solution. This leads to an overall increase in the trans-membrane flux, indicating better vapor mass transfer performance. In addition, the authors indicated that lithium-based solutions are suitable, as cations with fewer charges and smaller volumes improve membrane distillation performance. In Figure 9 and Figure 10, it can clearly be observed that the distilled water flux of H_2_O/LiBr was higher than that of the H_2_O/LiCl solution, but the H_2_O/LiBr + LiCl solution is in the middle.

Since LiCl exhibits properties as a long-term stability, lower cost and better hygroscopic performance [47], the ternary mixture H_2_O/LiBr + LiCl offers an alternative that could reduce the crystallization risk, the required mass transfer area, and the operational costs for sorption systems. In particular, low corrosivity and high salt solubility are desirable properties for long-term operation, as observed in previous tests with LiBr-based solutions, where membrane fouling by rust and salt depositions was noted [43]. This can lead to membrane wetting, reduced distilled water flux, and membrane degradation [48]. In addition, according to Arabi and Dehghani [22], the density of the H_2_O/LiBr + LiCl solution is lower than that of the LiBr aqueous solution; therefore, the costs associated with pumping could be lower as well and, if thermal renewable sources are used to power the AGMD, the economics of the process can be remarkably improved [34].

## 4. Conclusions

A membrane device with the AGMD configuration was assessed, operating with the H_2_O/LiBr + LiCl (with a mass ratio of 2:1, LiBr:LiCl) mixture, to evaluate the produced distilled water flux. To quantify the effect of the operating parameters (solution temperature, cooling water temperature, salt concentration, and membrane pore size), 64 experimental conditions were tested. The maximum distilled water flux was 7.63 kg/h·m^2^ with a solution temperature of 95.3 °C, a cooling water temperature of 25.1 °C, a salt concentration of 44.99% *w*/*w*, and a membrane pore size of 0.45 μm. On the other hand, the minimum distilled water flux was 0.28 kg/h·m^2^ at a solution temperature of 80.3 °C, a cooling water temperature of 40.1 °C, salt concentration of 50.05% *w*/*w*, and with a membrane pore size of 0.22 μm. Based on the results, solution temperature is the most significant parameter affecting the distilled water flux because membrane distillation is a thermally driven separation process. As the solution temperature increases, vapor mass transfer is improved; conversely, the effect of membrane pore size is slight. A comparison of the distilled water flux produced with the H_2_O/LiBr + LiCl solution with that of the conventional H_2_O/LiBr and H_2_O/LiCl solutions was conducted. The distilled water flux was higher with the H_2_O/LiBr solution than with the H_2_O/LiBr + LiCl solution, but its performance was still higher than that of the H_2_O/LiCl mixture. These results could lead to the development of new saline solutions for sorption systems based on membrane separation devices, providing a new background for desalinating non-conventional brines with membrane distillation.

## Figures and Tables

**Table 1:** Uncertainty of the measured variables.

Variable	Sensor/Instrument	Operation Range	Uncertainty
Temperature	RTD PT100	−30 to 350 °C	±0.1 °C
Cooling water volumetric flow	Volumetric flowmeter	0 to 8 L/min	±5.0% f.s. *
Heating fluid volumetric flow	Volumetric flowmeter	0 to 1.2 L/min	±4.0% f.s. *
Solution mass flow	Coriolis mass flowmeter	0 to 4.0 × 10^−2^ kg/s	±0.1%
Distillate water weight	Electronic balance	0 to 600 g	±0.01 g
Solution density	Coriolis mass flowmeter	0 to 5000 kg/m^3^	±0.5 kg/m^3^

*: f.s., full scale.

**Table 2:** Experimental operating conditions.

Parameter	Value
Membrane pore size (μm)	0.22
	0.45
Salt concentration (% *w*/*w*)	50.05 ± 0.05
	44.99 ± 0.03
Cooling water volumetric flow (L/min)	2.4 ± 0.40
Saline solution mass flow (kg/s)	4.00 × 10^−2^ ± 2.84 × 10^−5^
Saline solution temperature (°C)	95.3 ± 0.1
	90.3 ± 0.1
	85.3 ± 0.1
	80.3 ± 0.1
Cooling water temperature (°C)	40.1 ± 0.1
	35.1 ± 0.1
	30.1 ± 0.1
	25.1 ± 0.1

**Table 3:** Experimental operating conditions for the H_2_O/LiBr solution [30].

Parameter	Value
Membrane pore size (μm)	0.22
Salt concentration (% *w*/*w*)	49.61 ± 0.07
Cooling water volumetric flow (L/min)	2.0 ± 0.35
Saline solution mass flow (kg/s)	4.00 × 10^−2^ ± 2.44 × 10^−5^
Saline solution temperature (°C)	95.2 ± 0.1
	90.2 ± 0.1
	85.3 ± 0.1
	80.2 ± 0.1
Cooling water temperature (°C)	40.1± 0.1
	35.1 ± 0.1
	30.1 ± 0.1

**Table 4:** Experimental operating conditions for the H_2_O/LiCl solution [31].

Parameter	Value
Membrane pore size (μm)	0.22
Salt concentration (% *w*/*w*)	41.05 ± 0.03
Cooling water volumetric flow (L/min)	2.0 ± 0.35
Saline solution mass flow (kg/s)	3.50 × 10^−2^ ± 1.83 × 10^−5^
Saline solution temperature (°C)	90.2 ± 0.1
	85.3 ± 0.1
	80.2 ± 0.1
Cooling water temperature (°C)	40.1± 0.1
	35.1 ± 0.1
	30.1 ± 0.1
	25.1± 0.1

## Data Availability

The data recorders presented in this study are available on request from the corresponding author.

## Abbreviations

The following abbreviations are used in this manuscript:

AGMD	Air Gap Membrane Distillation
CFCs	Chlorofluorocarbons
COP	Coefficient of Performance
HCFCs	Hydrochlorofluorocarbons
LEP	Liquid Entry Pressure
MD	Membrane Distillation
PHE	Plate Heat Exchanger
PP	Polypropylene
PVDF	Polyvinylidene difluoride
RO	Reverse Osmosis
RTD	Resistance Temperature Detector
LiBr	Lithium Bromide
LiCl	Lithium Chloride
